# Pharmacist-led intervention in treatment non-adherence and associated direct costs of management among ambulatory patients with type 2 diabetes in southwestern Nigeria

**DOI:** 10.1186/s12913-021-06979-z

**Published:** 2021-09-22

**Authors:** Aduke E. Ipingbemi, Wilson O. Erhun, Rasaq Adisa

**Affiliations:** 1 Department of Clinical Pharmacy and Pharmacy Administration, Faculty of Pharmacy, University of Ibadan, Ibadan, Oyo state Nigeria; 2 Department of Clinical Pharmacy and Pharmacy Administration, Faculty of Pharmacy, Obafemi Awolowo University, Ile-Ife, Osun state Nigeria

**Keywords:** Pharmacist’s intervention, Treatment non-adherence, Type 2 diabetes, Direct costs of management, Nigeria

## Abstract

**Background:**

Non-adherence to recommended therapy remains a challenge to achieving optimal clinical outcome with resultant economic implications.

**Objective:**

To evaluate the effect of a pharmacist-led intervention on treatment non-adherence and direct costs of management among patients with type 2 diabetes (T2D).

**Method:**

A quasi-experimental study among 201-patients with T2D recruited from two-tertiary healthcare facilities in southwestern Nigeria using semi-structured interview. Patients were assigned into control (HbA1c < 7%, *n* = 95) and intervention (HbA1c ≥ 7%, *n* = 106) groups. Baseline questionnaire comprised modified 4-item Medication Adherence Questions (MAQ), Perceived Dietary Adherence Questionnaire (PDAQ) and International Physical Activity Questionnaire, to assess participants’ adherence to medications, diet and physical activity, respectively. Post-baseline, participants were followed-up for 6-month with patient-specific educational intervention provided to resolve adherence discrepancies in the intervention group only, while control group continued to receive usual care. Subsequently, direct costs of management for 6-month pre-baseline and 6-month post-baseline were estimated for both groups. Data were summarized using descriptive statistics. Chi-square, McNemar and paired t-test were used to evaluate categorical and continuous variables at *p* < 0.05.

**Results:**

Mean age was 62.9 ± 11.6 years, and 160(79.6%) were females. Glycated haemoglobin (HbA1c) was 6.1 ± 0.6% (baseline) and 6.1 ± 0.8% at 6-month post-baseline (*p* = 0.094) for control group, and 8.7 ± 1.5% (baseline) versus 7.8 ± 2.0% (6-month), *p* < 0.001, for the intervention. Post-baseline, response to MAQ items 1 (*p* = 0.017) and 2 (*p* < 0.001) improved significantly for the intervention. PDAQ score increased significantly from 51.8 ± 8.8 at baseline to 56.5 ± 3.9 at 6-month (*p* < 0.001) for intervention, and from 56.3 ± 4.0 to 56.5 ± 3.9 (*p* = 0.094) for the control group. Physical activity increased from 775.2 ± 700.5 Metabolic Equivalent Task (MET) to 829.3 ± 695.5MET(*p* < 0.001) and from 901.4 ± 743.5MET to 911.7 ± 752.6MET (*p* = 0.327) for intervention and control groups, respectively. Direct costs of management per patient increased from USD 327.3 ± 114.4 to USD 333.0 ± 118.4 (*p* = 0.449) for the intervention, while it decreased from USD 290.1 ± 116.97 to USD289.1 ± 120.0 (*p* = 0.89) for control group, at baseline and 6-month post-baseline, respectively.

**Conclusion:**

Pharmacist-led intervention enhanced adherence to recommended medications, diet and physical activity among the intervention patients, with a corresponding significant improvement in glycaemic outcome and an insignificant increase in direct costs of management. There is a need for active engagement of pharmacists in management of patients with diabetes in clinical practice.

**Trial registration:**

ClinicalTrials.gov identifier: NCT04712916. Retrospectively-registered.

## Background

Diabetes is one of the common non-communicable diseases worldwide [1]. The World Health Organisation (WHO) has reported that the prevalence of diabetes is rapidly increasing in low- and middle-income countries (LMICs) [2]. In Africa, 14.7 million adults are estimated to have diabetes, with Nigeria having the largest number of people with diabetes in Africa [3, 4]. The WHO estimates that about 1.7 million people are living with diabetes in Nigeria, which is expected to increase to 4.8 million by the year 2030 [4–6]. About one-quarter to one-third of all hospitalisation in medical (nonsurgical) wards in Nigeria have been linked to diabetes and its associated complications [7, 8]. Generally, in Nigeria, patients with diabetes either in ambulatory or institutionalised care typically make out-of-pocket payment for all their treatment expenses. There is no subsidy provision for the general populace as at present. However, few employees from organised private sector or government-owned public institutions, who might have enrolled under the National Health Insurance Scheme (NHIS) are required to pay only 10% of the total costs of treatment covering mostly the prescribed medications and laboratory investigations. In addition, both the secondary and tertiary care facilities are usually involved in treatment and care for diabetes patients, but comprehensiveness of management received by the patients may relatively differ between the two tiers, especially in relation to the diverse medical specialties and higher number of medical consultants in the tertiary hospitals.

In many LMICs including Nigeria, patients with diabetes faced varying challenges, ranging from lack of access to adequate medical facilities, socio-economic problems, to experience of fluctuating magnitude of disease complications [9, 10]. However, the goal of management for diabetes requires optimal adherence to recommended therapies, in order to achieve optimal clinical outcomes, and subsequently a reduced healthcare cost [11, 12]. In many developed and developing countries, it is estimated that about 50 to 60% of patients with chronic diseases including diabetes are non-adherent to prescribed therapies [13–15]. Specifically, in most developing countries, poor adherence to prescribed therapies among ambulatory patients with diabetes is a growing concern for healthcare providers and patients [15–17], partly because of its adverse consequences on therapeutic outcomes [18–20]. Non-adherence is especially high among patients with chronic diseases including diabetes mellitus largely because they require long-term and sometimes complex treatment regimen to control symptoms and prevent complications [17, 21]. However, suboptimal adherence to prescribed diabetes medications has been reported to account for 30 to 50% of treatment failure and worsen treatment outcomes with the attendant complications [9, 10]. Previous studies have also reported that patients with diabetes who are non-adherent had both statistically and clinically worse outcomes than their adherent counterparts [22, 23]. In addition, poor glycaemic control among patients with diabetes is associated with reduced treatment benefits, as well as increased financial burden on both patients and the society [24–26]. The challenge of treatment non-adherence among these patients may perhaps be averted if patients are adequately counseled on the necessity for optimal commitment to prescribed therapies, while non-adherent behaviours appropriately resolved [27–30]. Studies in many developed countries have reported improvement in medication adherence and clinical outcomes in pharmacist-conducted medication management among patients with diabetes [29, 31–33]. Also, it has been shown that a high level of medication adherence is associated with lower disease-related medical costs [12, 34]. The more patients adhered to therapy, the better the achievement of good clinical outcome, and the less is the length of hospitalisation and costs of management [12, 35–37].

Although, studies in some developed countries have related poor glycaemic control to higher healthcare costs [36–38]. Also, there had been studies conducted in LMICs including Nigeria to explore medication adherence and glycaemia among ambulatory patients with diabetes [17, 14, 25, 39], and a few on adherence to physical activity and dietary recommendations [40, 41]. However, none of these studies from developing countries comprehensively explore the interplay between treatment adherence, clinical outcomes and associated costs of management among patients with type 2 diabetes. This study therefore employed validated tools to evaluate therapy adherence and patient-specific reasons for non-adherence among ambulatory patients with type 2 diabetes in two tertiary hospitals, who had good glycaemia (HbA1c < 7%; control group) and those with poor glycaemia (HbA1c ≥ 7%; intervention group). We also evaluated the effect of pharmacist-led educational intervention in resolving identified adherence discrepancies on medications, diet and physical activity among patients in the intervention group only. Direct costs of management including transportation, consultation, medications and laboratory investigations for 6-month pre-baseline and 6-month post-baseline were subsequently estimated for both groups.

## Method

### Study site

University College Hospital (UCH) Ibadan, Oyo State and Federal Medical Centre (FMC) Abeokuta, Ogun State. The UCH is a 900-bed teaching hospital and is affiliated with University of Ibadan, Oyo state, Nigeria. The FMC is a 350-bed hospital and serves as a teaching hospital for BABCOCK University in Ogun state, Nigeria.

### Study design

A quasi-experimental study among T2D patients recruited from the two hospitals using questionnaire-guided semi-structured interview. At baseline, participants with HbA1c ≥7% (poor glycaemia) were assigned into intervention group, while those with HbA1c < 7% (good glycaemia) were considered as control group. Post-baseline, patient-specific pharmacist-led educational intervention was provided for participants in the intervention group only, to resolve adherence discrepancies in medications, diet and physical activity. The control group continued to receive usual care. At the end of 6-month post-baseline, the same questionnaire used for baseline interview was re-administered to participants in both groups to ascertain the extent of change in the measured variables at baseline. In this study, the direct costs of management defined as the sum of transport fare, consultation fee, as well as costs of medications and laboratory investigations were calculated 6-month pre-baseline and 6-month post-baseline for individual patient. The 6-month pre-baseline costs of management was estimated using key parameters garnered retrospectively from individual patient’s case note. This included date and number of clinics attended within 6-month prior to the baseline enrolment, details of prescribed medications comprising dosage form, drug name, dosage strength, frequency and duration of use, as well as diabetes-specific laboratory investigations. Total cost of transport fare was estimated by multiplying the number of clinics attended within the period by the prevailing transport cost, as indicated by each patient. The National Union of Road Transport Workers (NURTW) recommended fare for commercial/public vehicles was used as a guide. Patients were courteously asked about the rate of transport fare to the hospital during the prospective baseline interaction, while the amount/cost indicated was used to multiply the number of clinics attended within the 6-month pre-baseline. Consultation fee was calculated using the hospital’s approved fee which remained relatively stable in both hospitals within the period. Cost of medications was calculated using the price value of each medication from each hospital pharmacy unit, taking into consideration the daily dosage and duration of therapy. Also, the cost of laboratory investigations was estimated using the approved price from each hospital laboratory. Similar information was garnered and the same procedure followed to estimate the direct costs of management for the 6-month post-baseline.

### Study population

Adult T2D patients attending the endocrinology out-patient clinic of each hospital.

### Inclusion and exclusion criteria

Adult out-patients with primary diagnosis of T2D, who must have also been on antidiabetes medications for at least 6-month prior to the commencement of the study. Patients with Type 1 diabetes, gestational diabetes and T2D who declined participation were excluded.

### Sample size determination

Average of 25 patients with T2D regularly attended the weekly medical out-patient endocrinology clinic of each hospital. This gave a total of 100 patients per month in each hospital, which translated to an estimated population of 600 T2D per hospital for the 6-month study period. However, information obtained from the medical record unit of each hospital indicated that a maximum of 2 to 6 months clinic appointment is usually given to T2D in UCH, and a maximum of 3 months appointment in FMC, depending on the extent of glycaemic control. Based on this information, and considering the regular attendees of 100 T2D patients per month in each hospital, a total of 700 T2D (400 in UCH, and 300 in FMC) was considered as estimated population to guide the calculation of sample size. Thus, using the estimated population, at 95% confidence level and 5% margin of error, the Raosoft® sample size calculator [https://www.raosoft.com/samplesize.html] gave a value of 248. However, addition of 10% attrition rate gave a target sample size of 272.8 (rounded off to 273) to guide enrolment of participants.

### Sampling and recruitment procedure

**Fig. 1:**
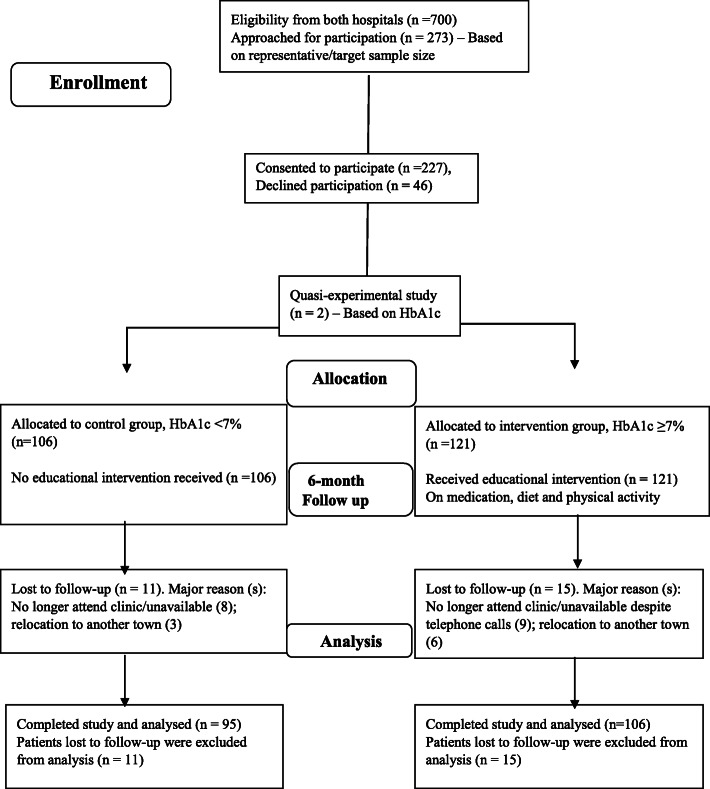
CONSORT flow diagram for participants’ enrolment

On every diabetes clinic day of each hospital, the principal investigator first checked and screened the medical records/case notes of T2D attendees for eligibility. Eligible case notes were consecutively selected and individual patient’s hospital number was used as identification tag/code. On each clinic, eligible patients were called by their respective hospital number which is always written on each patient’s small hospital appointment card. This was done to identify the precise location of each patient while waiting for their turn of physician consultation. Subsequently, patients were approached individually to introduce the study, as well as informed them of the purpose and procedures for involvement in the study. These were clearly explained to individual patient generally in English, and specifically in the local language (Yoruba). The informed consent form as approved by the Institution Review Board (IRB) was given to individual patient to read, while clarifications were made when necessary. Elderly patients were assisted by caregivers who accompanied them to the hospital. The instrument and informed consent form were translated into Yoruba, the predominant local language of participants, while back-translation was subsequently done to ensure response consistency. Patients were individually asked to indicate their intention to participate in the study by appending written signature or thumbprint in the appropriate space provided on the informed consent form. This approach was consistently followed and assured throughout the recruitment period at baseline. Patients were assured of their anonymity and confidentiality of response and were also told that their participation in the study is voluntary. Only the eligible patients who gave voluntary informed consent were enrolled, while those who declined participation were excluded from the study. At baseline, 273 patients were approached, while 227 (83.2%) consented to partake from both hospitals within the study period. Questionnaire was administered to consented participants and diabetes-specific clinical parameters, precisely HbA1c and blood pressure (BP) were also assessed. Each participant had individualised baseline, while intervention continued for patients in the intervention group by the principal investigator. Once the 6-month duration of involvement in the study is completed, then individual participation was considered terminated. At the end of 6-month follow-up period, a total of 201 (88.5%) patients completed the study from both hospitals and were those considered for data analysis. This comprised 95 patients with good glycaemia (HbA1c < 7%; control group) and 106 patients with poor glycaemia (HbA1c ≥7%; intervention group) using the ADA target for diabetes control [42]. Twenty-six (11.5%) participants were lost to follow-up including 11 patients from control group and 15 in the intervention group. Detail of participants’ enrolment is shown in the CONSORT flow diagram/chart (Fig. 1).

Finger pin-prick blood assay of glycated haemoglobin (HbA1c) was done using point-of-care kit, while BP measurements using Omron® digital monitor were taken on two separate occasions at few minutes interval and the average value was recorded. Patients’ BP and HbA1c were also rechecked after 6-month of interaction to assess the extent of change in the diabetes-specific clinical parameters.

### Data collection instrument

The baseline questionnaire comprised sections A to F. Section A captured demographic data, including monthly income and mode of medical bill payment. Section B contained modified International Physical Activity Questionnaire short-form (IPAQ-SF) [43], with individual physical activity calculated as metabolic equivalent task (MET) per week [44]. Section C consisted of modified Perceived Dietary Adherence Questionnaire (PDAQ) developed by Assad et al [45] to measure patients’ perception of dietary adherence. For these items, higher scores reflected higher level of adherence [45]. In this study, participants were classified as adherent to dietary recommendations if they score 51 (80%) and above out of the 63 maximum obtainable score, while those who scored less than 51 (80%) were classified as dietary non-adherent. Section D contained modified 4-item Medication Adherence Questions (MAQ). A ‘No’ response to all the 4-item MAQ was considered as optimal (100%) commitment to prescribed medication(s), while a ‘Yes’ response to any of the 4-item questions suggested suboptimal (< 100%) commitment to medication-taking [15, 46, 47]. Section E contained open-ended questions which explored reason(s) for non-adherence to medications, physical activity and dietary recommendations. Section F contained components of educational intervention to resolve adherence discrepancies identified in the response to question-items in MAQ, IPAQ-SF and PDAQ among patients in the intervention group. Where necessary, at least one or two clinic appointments were rescheduled to follow-up the participants in the intervention group. The 6-month post-baseline evaluation employed the same question-items in sections B, C, D and E to re-assessed participants in both control and intervention groups, with a view to ascertain the possible change in adherence status to recommended medications, diet and physical activity.

### Validation and pre-test of questionnaire

The questionnaire was assessed for content validity by an endocrinologist in UCH and three pharmacists in the academia who were knowledgeable about diabetes mellitus. A pre-test was done among twenty-five randomly selected T2D patients in UCH who were subsequently excluded from the main study. Patients for the pre-test were chosen from UCH alone largely in consideration of a relatively high patronage of patients with diabetes when compared to FMC. Specifically, the adapted IPAQ-SF [43] and PDAQ [45] scales were further re-evaluated/validated among the pre-test participants, in order to ensure reproducibility of the item-statements in the scales, as well as its effective use among the Nigerian patients. Feedback from the pre-test led to modifications in the instrument including some questions on physical activity which were rephrased as open-ended questions. In addition, some food items in the PDAQ that are not native food of the studied population were replaced with their indigenous food with similar calorie in accordance with official recommendations [48, 49]. However, these changes did not affect the scoring and the validity of the scales. Internal consistency of the question-items in PDAQ and MAQ was subsequently determined using Cronbach alpha test with values of 0.87 and 0.76, respectively.

### Educational intervention provided for participants in the intervention group

The face-to-face patient-specific educational intervention for participants in intervention group was provided by the principal investigator in the endocrinology clinic of each hospital. The intervention largely focused on clarification of medication doses, time of use, side effects as well as measure(s) to overcome some of the identified challenges including missed doses, types and kinds of foods, calorie intake, appropriate time of medication use in relation to meal, salt restriction, spacing of meal time and storage of food especially fruits. Prioritising tolerable physical activity was also emphasized. The educational intervention provided for patients in the intervention group was largely verbal, but was supplemented by a purposely designed educational resource material given to the intervention patients at the baseline recruitment stage, as a form of guidance for dietary recommendations and physical activity. The resource material basically contained the necessary tips on preferable types of food, fruits and vegetables, as well as the most useful forms of physical activity for patients with diabetes, such as brisk walking. The rescheduled appointment(s) of at least once or twice for participants in the intervention group within the 6-month post-baseline follow-up, allowed for reinforcement of the importance of positive adherence behaviours to achieve better therapeutic outcome. It also helped in ascertaining the extent of uptake of counselling tips/advice on the knowledge gaps identified during the baseline interaction. This approach was consistently maintained for patients in the intervention group, while those in the control group continued to receive usual care. Participants’ response to different sections of the questionnaire at baseline and within the 6-month follow-up was captured and documented in the coded questionnaire for individual patient. The intervention was provided by the principal investigator based on previous professional practice experience and clinical knowledge in diabetes management, while other co-investigators monitored the study progress and the intervention process. This was in a bid to ascertain strict compliance with the study protocol, while ensuring immediate and consistent entering of data as collected. Also, the intervention was carried out following the Template for Intervention Description and Replication (TIDER) checklist [50].

### Data analysis

Data were entered into SPSS (version 23), with descriptive and inferential statistics employed for analysis. Paired t-test was used to evaluate the extent of change in continuous variables, especially the clinical parameters, IPAQ, PDAQ and cost variables at baseline and 6-month post-baseline. McNemar test was used to assess for significant change in the response to MAQ items at baseline and 6-month post-baseline. Chi-square (χ^2^) was used to test for association between categorical variables (gender, age, educational qualifications) and baseline glycaemia, as well as adherence status at *p* < 0.05.

## Results

### Demographic and clinical characteristics of participants

Table 1 shows the demographic and clinical characteristics of participants. There were more females (160; 79.6%) compared to males (41; 20.4%). Mean age was 62.9 ± 11.6 years, and average monthly income was USD 189.3 ± 253.6 (Table 1). The mean HbA1c for the control group at baseline and at 6-month were 6.1 ± 0.6% and 6.1 ± 0.8%, respectively, while the mean HbA1c for the intervention group were 8.7 ± 1.5% and 7.8 ± 2.0% at baseline and 6-month post-baseline, respectively. Associations between demographic characteristics and baseline HbA1c values for participants in both groups are: gender (χ^2^ = 11.381; *p* = 0.001), age (χ^2^ = 5.72; *p* = 0.125), and educational qualification (χ^2^ = 2.781; *p* = 0.427). Also, relationship between demographic variables and baseline medication adherence of participants included age (χ^2^ = 3.554; *p* = 0.169), gender (χ^2^ = 0.486; *p* = 0.486) and educational qualification (χ^2^ = 4.155; *p* = 0.245).

**Table 1:** Demographic and clinical characteristics of participants

Demographics/clinical characteristics	UCH (*n* = 126)	FMC (*n* = 75)	Total (*n* = 201)
	Frequency (%)	Frequency (%)	Total (%)
Gender			
Male	31 (24.6)	10 (13.3)	41 (20.4)
Female	95 (75.4)	65 (86.7%)	160 (79.6)
Age (year)			
Mean	64.9 ± 11.36	59.6 ± 11.41	62.9 ± 11.6
Educational qualification			
No formal education	20 (15.9)	14 (18.7)	34 (16.9)
Primary	38 (30.2)	17 (22.7)	55 (27.4)
Secondary	21 (16.7)	14 (18.7)	35 (17.4)
Tertiary	47 (37.3)	30 (40.0)	77 (38.3)
Occupation			
Retiree	64 (50.8)	18 (24.0)	82 (40.8)
Self-employed (Artisan, petty traders, remittance, Other self-engaged jobs)	41(32.5)	37 (49.3)	78 (38.8)
Civil servant	8 (6.3)	12 (16.0)	20 (10.0)
Unemployed	11 (8.7)	5 (6.7)	16 (79.6)
Employed in a private firm	2 (1.6)	3 (4.0)	5 (2.5)
Marital status			
Married	112 (88.9)	72 (96.0)	184 (91.5)
Divorce	0 (0.0)	0 (0.0)	0 (0.0)
Widow/widower	14 (11.1)	3 (4.0)	17 (8.5)
Monthly income (USD)			
< 66.0	25 (19.8)	10 (13.3)	35 (17.4)
66.0–165.0	63 (50.0)	32 (42.7)	45 (47.3)
165.1–247.5	17 (13.5)	13 (17.3)	30 (14.9)
247.6–330.0	5 (4.0)	5 (6.7)	10 (5.0)
> 330.0	16 (12.7)	15 (20.0)	31 (15.4)
Mean monthly income ±SD (USD)	164.9 ± 214.6	230.3 ± 305.4	189.3 ± 253.5
Year of diagnosis			
Mean ± SD	11.4 ± 8.95	6.75 ± 7.21	9.65 ± 8.62
< 5	33 (36.2)	42 (56.0)	75 (37.3)
5–9	23 (18.3)	11 (14.7)	34 (16.9)
≥ 10	70 (55.6)	22 (29.3)	92 (45.8)
Family history of hypertension			
Yes	52 (41.3)	21 (28.0)	73 (36.3)
No	45 (35.7)	24 (32.0)	69 (34.3)
Don’t know	29 (23.0)	30 (40.0)	59 (29.4)
Family history of DM			
Yes	27 (21.4)	24 (32.0)	51 (25.4)
No	60 (47.6)	20 (26.7)	80 (39.8)
Don’t know	39 (31.0)	31 (41.3)	70 (34.8)

USD = United States of America Dollar, SD = Standard Deviation, UCH = University College Hospital, FMC = Federal Medical Centre, DM = Diabetes Mellitus, 1USD = ₦303

### Participants’ response to MAQ, PDAQ and IPAQ at baseline and 6-month post-baseline

Overall, at baseline, 65 (68.4%) of patients in the control and 45 (42.5%) in the intervention responded ‘No’ to all the 4-items in MAQ scale, indicating optimal (100%) commitment to medication-taking as prescribed. However, at the end of 6-month post-baseline, 75 (78.9%) of the patients in the control and 80 (75.5%) in the intervention had optimal commitment to their medication(s). Detail of participants’ response to MAQ is shown in Table 2. Evaluation of PDAQ scale indicated that, at baseline, 84 (88.4%) in the control and 67 (63.2%) in the intervention had score ≥ 51 (i.e. dietary adherent), while at 6-month post-baseline, 87 (91.6%) in the control and 95 (89.6%) in the intervention were dietary adherent (Table 3). The IPAQ evaluation showed that, a total of 59 (62.1%) and 64 (60.1%) patients in the control and intervention groups, respectively had ≥600MET physical activity per week at baseline (i.e. adherence to recommended minimum physical activity). Whereas, 66 (69.5%) in the control and 76 (71.7%) in the intervention were adherent to recommended physical activity at 6-month post-baseline (Table 3).

**Table 2:** Response of participants to the 4-item Medication Adherence Questions (MAQ) at baseline and 6-month post-baseline

Items	Control (n = 95)				Mc Nemar test	Intervention (*n* = 106)				Mc Nemar test
	Baseline		6-month post baseline		*p*-value	Baseline		6-month post baseline		p-value
	Yes n(%)	No n (%)	Yes n (%)	No n(%)		Yes n(%)	No n(%)	Yes n(%)	No n(%)	
1. Are there times when you forget to take your DM/HHDx medicine(s)?	8 (8.4)	88 (92.6)	5 (5.3)	90 (94.7)	0.219	15 (14.2)	91 (85.8)	8 (7.5)	98 (92.5)	0.017*
2. Do you have problems remembering to take your DM/HHDx medication(s) in past few weeks?	17 (17.9)	78 (82.1)	14 (14.7)	91 (95.8)	0.607	36(34.0)	70 (66.0)	12(11.3)	94 (88.7)	0.000*
3. Are there times when you feel much better and you discontinue your DM/HHDx medicine(s)?	4 (4.2)	91 (95.8)	1 (1.1)	94 (98.9)	0.25	5 (4.7)	101 (95.3)	2 (1.9)	104 (98.1)	1.00
4. Are there times when you feel uncomfortable with your medicine(s) and you stop taking them?	3 (3.2)	92 (96.8)	4 (4.2)	91 (95.8)	1.00	4 (3.8)	102 (96.2)	2 (1.9)	104 (98.1)	0.687

HHDX: Hypertensive Heart Disease; Control group - HbA1c < 7.0%; Intervention group - HbA1c ≥ 7%. A ‘No’ response to all the 4-item questions was considered as total (100%) commitment to medication-taking/adherence, while a ‘Yes’ response to any of the 4-item questions indicated suboptimal (< 100%) adherence. *Significant difference with McNemar test, Level of significant *p* < 0.05

**Table 3:** Clinical outcomes and adherence parameters at baseline and 6-month post-baseline

ITEMS		UCH (n = 126)		p- value	FMC (n = 75)		p-value	Total (201)		p-value
	Group	Baseline n (%)	6-month post baseline n (%)		Baseline n (%)	6-month post-baseline n(%)		Baseline n (%)	6-month post baseline n (%)	
MAQ	Control < 100%	22 (31.4)	13 (18.6)	0.04^+^	8 (32.0)	7(28.0)	0.18^+^	30 (31.6)	20 (21.1)	0.041^+^
	Control = 100%	48 (68.6)	57 (81.4)		17 (68.0)	18(72.0)		65 (68.4)	75 (78.9)	
	Intervention < 100%	36 (64.3)	15 (32.1)	0.00^+^	25 (50.0)	11 (32.0)	0.035^+^	61 (57.5)	26 (24.5)	0.000^+^
	Intervention = 100%	20 (35.7)	41(73.2)		25 (50.0)	39 (78.0)		45 (42.5)	80 (75.5)	
IPAQ (MPW)	Control <600MPW	27 (38.6)	23 (32.9)	0.00^+^	9 (36.0)	6 (24.0)	0.453^+^	36 (37.9)	29 (30.5)	0.98^+^
	Control ≥600MPW	43 (61.1)	47 (67.1)		16 (64.0)	19 (76.0)		59 (62.1)	66 (69.5)	
	Intervention <600MPW	17 (30.4)	11 (19.6)	0.07^+^	25 (50.0)	19 (38.0)	0.18^+^	42 (39.6)	30 (28.3)	0.00^+^
	Intervention ≥600MPW	39 (69.6)	45 (80.4)		25 (50.0)	31 (62.0)		64 (60.4)	76 (71.7)	
IPAQ (MET) Mean ± SD	Control	945.2 ± 842.9	977.1 ± 853.4	0.002^*^	777.1 ± 312.1	729.2 ± 291.4	0.161^*^	901.4 ± 743.5	911.7 ± 752.6	0.327^*^
	Intervention	1058.4 ± 935.6	1102.5 ± 908.6	0.001^*^	552.1 ± 224.7	608 ± 244.0	0.005^*^	775.2 ± 700.5	829.3 ± 695.5	0.000^*^
PDAQ Diet scale	Control < 51	8 (11.4)	7 (10.0)	1.00^+^	3 (12.0)	3 (12.0)	1.00^+^	11 (11.6)	8 (8.4)	0.45.3^+^
	Control ≥51	62 (88.6)	63 (90.0)		22 (88.0)	22 (88.0)		84 (88.4)	87 (91.6)	
	Intervention < 51	26 (46.4)	7 (12.5)	0.000^+^	13 (26.0)	2 (4.0)	0.001^+^	39 (36.8)	11 (10.4)	0.000^+^
	Intervention ≥51	30 (53.6)	49 (87.5)		37 (74.0)	48 (96.0)		67 (63.2)	95 (89.6)	
PDAQ Mean ± SD	Control	56.4 ± 4.0	56.6 ± 3.8	0.094^*^	55.9 ± 4.1	56.3 ± 4.2	0.44^*^	56.3 ± 4.0	56.5 ± 3.9	0.094^*^
	Intervention	50.3 ± 10.1	56.3 ± 4.4	0.000^*^	53.4 ± 6.7	56.5 ± 4.1	0.00^*^	51.8 ± 8.8	56.4 ± 4.3	0.000^*^
HbA1c (%)	Control < 7%	70 (55.6)	102 (81.0)	1.00^+^	25 (33.3)	32 (42.7)	0.016^+^	95 (47.3)	134 (66.7)	0.008^+^
	Intervention ≥7%	56 (44.4)	24 (19.0)	0.000^+^	50 (66.7)	43 (57.3)	0.000^+^	106 (52.7)	67 (33.3)	0.000^+^
HbA1c Mean ± SD (%)	Control	6.1 ± 0.6	6.0 ± 0.7	0.005^*^	6.4 ± 0.5	6.7 ± 1.0	0.15^*^	6.1 ± 0.6	6.1 ± 0.8	0.094^*^
	Intervention	8.4 ± 1.3	7.0 ± 1.3	0.00^*^	9.0 ± 1.7	8.8 ± 2.3	0.492^*^	8.7 ± 1.5	7.8 ± 2.0	0.000^*^
**S**BP (mmHg) Mean ± SD	Control	127.3 ± 16.9	122.7 ± 13.6	0.002^*^	134.2 ± 20.9	132.4 ± 19.2	0.574^*^	129.3 ± 18.2	125.3 ± 15.9	0.004^*^
	Intervention	134.3 ± 20.0	125.7 ± 19.3	0.000^*^	140.4 ± 21.1	137.1 ± 19.3	0.33^*^	136.9 ± 20.7	131.0 ± 19.9	0.002^*^
DBP (mmHg) Mean ± SD	Control	76.9 ± 8.6	74.3 ± 7.2	0.007^*^	77.3 ± 9.5	77.9 ± 9.5	0.814^*^	77.1 ± 8.93	75.3 ± 8.0	0.068^*^
	Intervention	80.35 ± 10.9	76.2 ± 7.2	0.009^*^	80.2 ± 9.9	80.1 ± 4.4	0.973^*^	80.2 ± 10.4	78.3 ± 9.6	0.074^*^

Pearson Chi-square of adherence status among patients showed more males with glycaemic control (HbA1c < 7%) compared to females (χ^2^ = 11.381, p = 0.001) after intervention

MAQ = Medication Adherence Questions; IPAQ- International Physical Activity Questionnaire; PDAQ = Perceived Dietary Adherence Questionnaire; MPW- Minimum Physical Activity Per Week measured in unit of MET; MET = Metabolic Equivalent Task; SBP = Systolic Blood Pressure; DBP = Diastolic Blood Pressure; HbA1c = Glycated haemoglobin; *Paired t-test, p < 0.05 significant; ^+^McNemar test, p < 0.05 significant

### Clinical outcomes and adherence parameters evaluated at baseline and 6-month post-baseline

In the intervention group, there was a significant reduction in the HbA1c from 8.7 ± 1.5% at baseline to 7.8 ± 2.0% at 6-month (*p* < 0.001). The systolic blood pressure reduced from 136.9 ± 20.7 mmHg at baseline to 131.0 ± 19.9 mmHg at 6-month (*p* = 0.002). In addition, there was a significant improvement in the average weekly physical activity from 775.2 ± 700.5MET at baseline to 829.3 ± 695.5MET at 6-month (p < 0.001) and a significant increase in adherence to dietary recommendations from 51.8 ± 8.8 at baseline to 56.4 ± 4.3 at 6-month (p < 0.001) Table 3.

In the control group, there was no significant change in the HbA1c value at baseline (6.1 ± 0.6%) and at 6-month post-baseline (6.1 ± 0.8%), *p* = 0.094, but a significant reduction in systolic blood pressure from 129.3 ± 18.2 mmHg (baseline) to 125.3 ± 15.9 mmHg (6-month post-baseline), *p* = 0.004. Also, there was insignificant increase in adherence to dietary recommendations (56.3 ± 4.0 to 56.5 ± 3.9; p = 0.094), as well as the weekly physical activity (901.4 ± 743.5 to 911.7 ± 752.6MET; *p* = 0.327), Table 3.

### Reason(s) for treatment non-adherence among participants

Fifty-one (53.7%) in the control and 104 (98.1%) in the intervention gave one or combination of reasons for medication non-adherence. Financial constraints [control (25; 49.0%), intervention (48; 46.1%)] and forgetfulness [control (16; 31.3%), intervention (36; 34.6%)] were mostly cited as major reason(s) for medication non-adherence (Table 4). Also, the reason(s) for non-adherence to dietary recommendations in different combination included: knowledge deficit of the importance of recommended diet for diabetes management [control (3; 18.8%), intervention (23; 38.3%)], financial constraints [control (3; 18.8%), intervention (11; 18.3%)], difficulty in accessing recommended diet [control (5; 31.3%), intervention (4; 6.7%)], lack of means of preservation e.g. refrigerator [control (5; 31.3%), intervention (14; 23.3%)], and inability to resist dietary desires [control (0; 0.0%), intervention (8; 13.3%)]. For the physical activity non-adherence, the reason(s) largely cited included: tiredness/discomfort [control (13; 36.1%), intervention (14; 33.3%)], lack of time/busy schedule [control (9; 25.0%), intervention (14; 33.3%)], unwillingness [control (8; 22.2%), intervention (8; 19.0%)] and illness [control (6; 16.7%), intervention (6; 14.3%)].

**Table 4:** Reasons for medication non-adherence among participants at baseline

Reason for medication non-adherence	T2D Participants	
	Control (%)	Intervention (%)
Financial constraint	25(49.0)	48 (46.1)
Forgetfulness	16 (31.3)	36 (34.6)
Symptom under control	3 (5.9)	3 (2.9)
Side effect(s)	4 (7.8)	4 (3.8)
Unavailability of prescribed medication(s)	2 (3.9)	1 (1.0)
Tired of medication use	0 (0.0)	7 (6.7)
I don’t know how to use it	0 (0.0)	2 (1.9)
I cannot read the instruction because of my eye defect	0 (0.0)	1 (1.0)
There is no consistency in the prescription of different physicians l am allotted to	1 (2.0)	2 (1.9)
**Total**	**51**	**104**

There were multiple responses among some participants

### Financial capacity and medical bill payment mechanism among participants

Of the 106 participants in the intervention group, 42 (39.6%) paid for medical bill by self via out-of-pocket (OOP), 34 (32.1%) had their caregivers responsible for paying OOP and 12 (11.3%) paid through co-OOP of caregivers with patients, while enrollees of National Health Insurance Scheme (NHIS) who usually pay 10% of their medical bill accounted for 11 (10.4%). Seven (6.6%) gave no response. In the control group, 44 (46.3%) paid medical bill by self OOP, 25 (26.3%) had it paid OOP by their caregivers, 14 (14.7%) co-OOP with caregivers, while 12 (12.6%) were NHIS enrollees.

### Change in direct costs of management for participants in the control and intervention groups

There was a significant decrease in mean costs per patient for consultation and transportation at 6-month post-baseline for both groups (Table 5). In the intervention group, the mean cost per patient for antidiabetes medications increased from an average of USD 117.3 ± 61.9 pre-baseline to USD 127.3 ± 66.2 at 6-month post-baseline (*p* = 0.025), while mean cost per patient for laboratory investigations decreased from USD 87.8 ± 28.1 to USD 82.3 ± 16.1 (*p* = 0.035). Overall, the mean direct costs of management per patient increased from USD 327.3 ± 114.4 (pre-baseline) to USD 333.0 ± 118.4 (6-month post-baseline), *p* = 0.449, in the intervention group. However, in the control group, the mean direct costs of management per patient decreased from USD 290.1 ± 117.0 (pre-baseline) to 289.1 ± 120.0 at 6-month post-baseline (*p* = 0.89), Table 5.

**Table 5:** Direct costs of management for participants in the control and intervention groups

Cost items	Group	Total cost		Mean cost per patient ± SD		Paired t-test	% increase/decrease in cost
		6-month pre-baseline (USD)	6-month post- baseline (USD)	6-month pre- baseline (USD)	6-month post baseline (USD)	p- value	
Consultation	Control (n = 95)	1400.7	1232.7	14.7 ± 6.5	13.0 ± 5.5	0.00^*^	12.0
	Intervention (n = 106)	1419.5	1300.7	13.4 ± 6.8	12.3 ± 6.4	0.001^*^	8.4
Transportation fare	Control (n = 95)	662.5	589.5	7.0 ± 6.7	6.2 ± 5.5	0.012^*^	11.0
	Intervention (n = 106)	1076.1	957.8	10.2 ± 11.2	9.0 ± 9.9	0.003^*^	11.0
Anti-hypertensives	Control (*n* = 72)	4218.3	4379.8	58.1 ± 47.1	60.8 ± 47.4	0.137	3.8
	Intervention (*n* = 80)	5137.4	5530.2	62.4 ± 55.9	68.8 ± 58.1	0.121	7.6
Antidiabetes medication(s)	Control (n = 95)	9521.9	9359.2	100.2 ± 59.3	98.5 ± 56.9	0.719	1.7
	Intervention (n = 106)	12,434.8	13,490.9	117.3 ± 61.9	127.3 ± 66.2	0.025^*^	8.5
Other medications	Control (*n* = 33)	3882.6	4458.8	104.1 ± 106.6	106.6 ± 74.5	0.678	14.8
	Intervention (*n* = 53)	5340.8	5290.3	94.0 ± 56.1	89.0 ± 67.4	0.589	0.9
Total medications	Control (n = 95)	17,589.8	18,192.8	185.2 ± 107.6	191.6 ± 114.5	0.345	3.4
	Intervention (n = 106)	22,912.9	24,311.4	216.2 ± 104.3	229.4 ± 128.5	0.068	6.1
Laboratory Investigations	Control (n = 95)	7906.4	7445.4	83.2 ± 25.4	78.4 ± 27.3	0.121	5.8
	Intervention (n = 106)	9308.9	8725.0	87.8 ± 28.1	82.3 ± 16.1	0.035^*^	6.3
Overall costs of management	Control (n = 95)	27,559.4	27,465.3	290.1 ± 117.0	289.1 ± 120.0	0.89	0.3
	Intervention (n = 106)	34,689.7	35,294.9	327.3 ± 114.4	333.0 ± 118.4	0.449	1.7

Percentage increase or decrease was calculated by subtracting cost at 6-month post-baseline from the cost at 6-month pre-baseline, divided by the cost at pre-baseline, then multiplied by 100. The cost for 6-month pre-baseline was calculated retrospectively from participants’ case notes using information on prescribed medications, laboratory investigations and number of clinic visit/consultations within 6-month prior to baseline enrolment, while patients’ cost of transportation to the hospital for current visit was used in calculating the transport fare. Mean cost per patient was calculated from the respective total cost divided by the corresponding number of patients. Total medications cost is the sum of the costs for antidiabetes, antihypertensive and other adjunct medications. Each hospital approved price for medication, laboratory investigation and consultation was used as a guide for calculating the respective cost

Conversion of Naira to USD as at January, 2017 was ₦303.0 to 1US dollar (https://www1.oanda.com/currency/converter/). ^*^Significant difference with paired t-test

## Discussion

In this study, we evaluated treatment adherence and patient-specific reason(s) for non-adherence among patients classified as good glycaemia (control group) and poor glycaemia (intervention group). This coupled with educational intervention provided for patients in the intervention group during a 6-month follow-up, while direct costs of management were subsequently estimated for both groups. Our study however showed a significant increase in the number of patients in the intervention group with optimal commitment to medication-taking at the end of 6-month post-baseline, compared to the control cohort. This may perhaps suggest that consistent verification of patients’ understanding and knowledge about a medical condition and its treatment during patient-provider’s encounter could be a key educational strategy to reveal the information gaps of patients [28]. Previous studies have also reported educational intervention to be most effective in resolving knowledge-related medication non-adherence behaviour [28, 51, 52]. In addition, our study reveals improvement in glycaemia in the intervention cohort, which may partly be linked to improved adherence to recommended therapies in the cohort compared to the control group. Though, the average HbA1c value in our study is above the American Diabetes Association (ADA) recommended target of < 7.0% [42], however, significant reduction (0.9%) in the average HbA1c from 8.7 ± 1.5% (baseline) to 7.8 ± 2.0% (6-month post-baseline) in the intervention group is consistent with previous studies in most developed and some developing countries [30, 32, 33]. Kiel et al in an observational prospective study to demonstrate the pharmacist’s impact on clinical outcomes in a diabetes disease management program showed an HbA1c reduction of 1.6% [53]. In addition, Chen et al reported a decrease of 0.83% in mean HbA1c in the intervention group during a randomised controlled trial on pharmaceutical care of elderly patients with poorly controlled T2D [38]. Also, Odegard et al in a randomised pharmacist’s intervention among poorly controlled diabetes mellitus reported a significant reduction in HbA1c from 10.2 to 8.7% after 6 months and 8.2% after 12 months [54]. It is also noteworthy to mention that, there is a corresponding increase in the proportion of patients in intervention group with good glycaemia at the end of 6-month post-baseline (66.7%) compared to 47.3% at baseline. Previous studies have indicated a strong correlation between increased therapy adherence and better glycaemic control among patients with T2D, with a possible reduction in the risk of diabetes complications, morbidity and mortality [28, 55, 56]. Also, the United Kingdom Prospective Diabetes Study Group has proved that for every 1% reduction in HbA1c, there is a 21% drop in the risk for any diabetes-related adverse events or complications, and a 21% reduction in deaths related to diabetes [57, 58]. The positive clinical outcome in the intervention group may therefore be indirectly linked to a consequent reduction in the risk of microvascular adverse events among the intervention patients. Thus, there may be the need for pharmacists, especially in LMICs to take cognizance of this study findings, by actively involved in holistic patient-centred adherence counseling for patients with diabetes in particular and chronic diseases in general, in a bid to ensure improved glycaemic outcome. This may become necessary if taking into consideration the inadequacies of the current process of adherence enhancement by physician alone [59, 60], where the information provided by physician about medication(s) might be insufficient for the patient, partly due to physician’s time constraints and high number of patients to attend to during clinic visits [60–62]. Pharmacists having direct contact with patients during filling and refilling of prescriptions have a better ability to detect potential or actual adherence problems, and having confirmed the existence of a non-adherence problem, intervene to resolve the actual problem(s) while preventing the development of potential ones [63].

Asides the reduction in glycaemic outcome among participants in the intervention group, we observed a significant increase of 8.5% in the mean cost per patient for antidiabetes medication(s) from USD 117.3 ± 61.9 to USD 127.3 ± 66.2, and a 6.1% increase in mean costs per patient for total medications. While the direct costs of management per patient changed from USD 327.3 ± 114.4 to 333.0 ± 118.4, indicating a 1.7% increase. However, in the control group, the mean cost per patient for antidiabetes medications decreased by 1.7%, while the total direct costs of management per patient decreased by 0.3%. Overall, the average direct costs of management for the intervention patients was USD 37.2 (11.4%) higher than that for patients in the control group. This seems consistent with Chen et al which reported the mean cost per patient of USD 44.10 in the intervention and USD 4.35 in the control group, representing an increase of USD 39.73 in cost per patient [38]. Sokol et al [64] and Roebuck et al [65] have reported that greater adherence to medication(s) for chronic condition is associated with higher medication costs but lower non-medical costs. It has also been shown that costs of care was significantly associated with glycaemic control [66, 67]**.** Mata-Cases et al [37] in their study reported that patients with diabetes who had poor glycaemic control (HbA1c > 7%) had increased costs of €448.0 per patient per year compared to those with good control (HbA1c ≤7%). The two main variables reported to cause the increase in costs in their study were medications and hospitalisation [37]. In our study, the increase in costs per patient for total medications in the intervention group may be partly attributed to poor clinical outcomes, which may necessitate aggressive management with therapy intensification for the cohort. Treatment intensification may include add-on medication(s) and sometimes brand substitution which may be relatively expensive than the generic equivalent, thereby contributing to higher costs of management [68–70]. Notwithstanding, healthcare providers including pharmacists may need to take up active role in educating patients on diabetes-specific treatment goals, as well as involving them in the treatment plans/decision geared towards achieving and maintaining target glycaemic outcome. This patient-centred approach to care may invariably lead to better outcome, with a possible reduction in the development of diabetes complications and by extension the costs of management.

The most common reasons for medication non-adherence among patients in both groups were forgetfulness and financial constraints. Forgetfulness has been identified as a major reason for non-adherence in previous studies [17, 28, 71]. Lack of financial coping mechanism has also been found to be an adherence barrier among patients with chronic illness [72, 73]. Patients who paid for their medication(s) out-of-pocket are more likely to adopt different costs-containment coping strategies such as erratic or irregular filling of prescriptions or taking less frequent doses to make the medication(s) last longer [73–75]. In addition, the topmost reason for dietary non-adherence among the cohorts was lack of knowledge on the importance of recommended diets for diabetes management. This is despite the fact that dietary management is an integral aspect of T2D management to achieve optimal glycaemic control [76]. Thus, in our study, identification of barriers to dietary adherence and the subsequent resolution of patient-specific dietary knowledge gaps in the intervention cohort, mostly helped in enhancing dietary self-care behaviour necessary to ensure optimal glycaemic outcome. Reduction in HbA1c value has been reported to be associated with ingestion of lower carbohydrate and low saturated fat diet in patients with diabetes [76–79]. Consumption of low glycaemic index carbohydrate and low cholesterol-containing diets are core dietary information consistently emphasized for the intervention patients. In addition, there is an increased number of patients in the intervention group with good response on adherence to physical activity at the end of 6-month post-baseline. This may perhaps be linked to increased awareness of positive impact of exercise on glycaemic control, as well as other health-related benefits of prescribed form of exercise. Educational intervention has been reported to improve patients’ participation in physical activity by changing their physical activity behaviour [80, 81]. However, tiredness and busy schedule were the most reported barriers to engagement in regular physical activity among the intervention patents, while unwillingness was most cited in the control group. Pati et al has reported unwillingness as one of the major barriers to increased participation in physical activity practice [82].

Despite the fact that our study findings serve as a useful evidence-based information to further reiterate the necessity for pharmacists’ active contribution in collaborative care of patients with diabetes generally. This study however, has the following limitations. The self-report nature of the tools used for data collection may be associated with some inherent bias such as recall bias, when patients may over- or under-report some of the information provided. Also, we employed quasi-experimental approach in which participants were solely assigned into control and intervention groups based on their respective glycated haemoglobin value. Thus, the possibility of the difference in baseline characteristics of participants affecting the eventual outcomes may not be totally ruled out. As a result, selection and channeling bias [83, 84] might be a concern in our study, since participants were not subjected to standard randomisation techniques that will assure equal chance of being allocated to either of the studied groups. Nevertheless, evaluation of association between the participants’ demographic characteristics and baseline HbA1c value, as well as medication adherence status indicated no statistically significant difference. Moreover, the scope and design of our study to categorize patients into ‘good’ and ‘poor’ glycaemia necessitate the use of quasi-experimental concept. Another limitation of our study may be linked to the recruitment of participants, as well as provision of intervention in the endocrinology clinic of the hospitals, which may perhaps have impacted on the emotion or psyche of some studied participants. Thus, the possibility of response bias may not be completely excluded. Nevertheless, the proactive and patient-centred measures adopted in our study including the courteous patient approach and consistent use of non-judgmental interacting skills during encounters might have assisted in boosting the morale of participants to provide an honest opinion/response. Also, the likelihood of selective outcome reporting and analysis bias may still be a concern, especially when the investigator serves as both the data collector and evaluator. This concern was partly allayed considering the adopted approach where the co-investigators are continuously monitoring the study progress and the intervention process to ensure strict compliance with the study protocol, while ensuring immediate and consistent entering of data as collected at each study site, with no propensity for conflicting data. In addition, the principal investigator, at intervals, always present the update on the data collected, where challenges encountered are highlighted and possible resolution proffered. Furthermore, in our study, the retrospective estimation of 6-month pre-baseline direct costs of management using the prevailing costs at the time of the study may not give a concise reflection of the pre-baseline cost value, on account of the time lag. Nevertheless, all the aforementioned limitations may need to be carefully considered, when interpreting the findings of our study, while caution should be exercised in making a widespread generalisation.

## Conclusion

Pharmacist-led intervention enhanced adherence to recommended medications, diet and physical activity among the poorly-controlled type-2-diabetes, with a corresponding significant improvement in glycaemic outcome and an insignificant increase in associated direct costs of management. This further underscores a need for proactive engagement of pharmacists in collaborative management of patients with type 2 diabetes, especially in respect to treatment adherence enhancement to achieve positive clinical outcomes.

## Data Availability

The data sets used and/or analysed during the current study are available from the corresponding author on request.

## Abbreviations

T2D: Type 2 diabetes mellitus
HbA1c: Glycated haemoglobin
MAQ: Medication Adherence Questions
PDAQ: Perceived Dietary Adherence Questionnaire
IPAQ: International Physical Activity Questionnaire
MET: Metabolic Equivalent Task
UCH: University College Hospital
FMC: Federal Medical Centre
USD: United States of America Dollar
UKPDS: United Kingdom Prospective Diabetes Study
NHIS: National Health Insurance Scheme
NURTW: National Union of Road Transport Workers
ADA: American Diabetes Association
CONSORT: Consolidated Standards of Reporting Trials
IRB: Institution Review Board
WHO: World Health Organisation
NREC: National Research and Ethic Committee
LMICs: Low and Middle Income Countries
